# Liver cirrhosis in children – the role of imaging in the diagnostic pathway

**DOI:** 10.1007/s00247-022-05480-x

**Published:** 2022-08-30

**Authors:** Jochen Herrmann, Philippe Petit, Enke Grabhorn, Alexander Lenz, Julian Jürgens, Stéphanie Franchi-Albella

**Affiliations:** 1 Section of Pediatric Radiology, Department of Diagnostic and Interventional Radiology and Nuclear Medicine, University Medical Center Hamburg-Eppendorf, Martinistrasse 52, 20251 Hamburg, Germany; 2 Aix Marseille Université, Hopital Timone-Enfants, Marseille, France; 3 Department of Pediatric Gastroenterology and Hepatology, University Medical Center Hamburg, Hamburg, Germany; 4 Department of Diagnostic and Interventional Radiology and Nuclear Medicine, University Medical Center, Hamburg, Germany; 5 Department of Pediatric Radiology, Hôpital Bicêtre, National Reference Centre for Rare Pediatric Liver Diseases, Paris, France

**Keywords:** Children, Cirrhosis, Computed tomography, Elastography, Imaging, Liver, Magnetic resonance imaging, Ultrasound

## Abstract

Liver cirrhosis in children is a rare disease with multifactorial causes that are distinct from those in adults. Underlying reasons include cholestatic, viral, autoimmune, hereditary, metabolic and cardiac disorders. Early detection of fibrosis is important as clinical stabilization or even reversal of fibrosis can be achieved in some disorders with adequate treatment. This article focuses on the longitudinal evaluation of children with chronic liver disease with noninvasive imaging tools, which play an important role in detecting cirrhosis, defining underlying causes, grading fibrosis and monitoring patients during follow-up. Ultrasound is the primary imaging modality and it is used in a multiparametric fashion. Magnetic resonance imaging and computed tomography are usually applied second line for refined tissue characterization, clarification of nodular lesions and full delineation of abdominal vessels, including portosystemic communications.

## Introduction

Cirrhosis is the common end point of chronic liver disease and a consequence of recurrent cellular damage with induction of diffuse fibrosis paralleled by nodular regeneration [1]. With progression of cirrhosis, scar tissue replaces normal liver parenchyma, which results in loss of hepatic function and portal hypertension over time. The clinical presentation depends on the stage of disease and whether manifestations are compensated. Some children with cirrhosis attract clinical attention with mild symptoms such as hepatomegaly, splenomegaly, elevated liver function tests or jaundice. Others present acutely ill with liver failure and signs of decompensation including ascites, variceal bleeding, pulmonary hypertension and, in some cases, hepatic encephalopathy [2]. Acute-on-chronic illnesses, especially, can result in rapid deterioration.

Despite a recently published population-based study from Canada that reported an increasing incidence of pediatric cirrhosis over the past two decades, liver cirrhosis in children is still rare [3]. A low index of suspicion in the usual clinical setting together with a long silent clinical course in many children explains why the diagnosis of liver cirrhosis is often delayed and only made after progressive parenchymal changes have already manifested. Nevertheless, advanced fibrosis or even cirrhosis in children is still considered a dynamic process that can be slowed, stopped or reversed if the underlying disease is adequately treated [1, 4, 5].

Among the most frequent diseases potentially resulting in cirrhosis in neonates and young children are biliary atresia and certain inherited metabolic disorders. In older children and adolescents, the main underlying diagnoses are autoimmune disorders, chronic viral hepatitis, Wilson disease, alpha-1-deficiency, cardiac causes and nonalcoholic fatty liver disease (NAFLD) [6].

Medical imaging techniques fulfill complex tasks in the management of patients with cirrhosis. At first presentation, imaging has a part in the initial diagnostic work-up, but the underlying diagnosis is often made based on clinical grounds incorporating laboratory findings, histopathology and genetic testing. Image-guided liver biopsy aids the diagnostic process with information on histology. Medical imaging methods have proven particularly useful for noninvasive evaluation of disease progression and identification of complications which directs treatment. Accordingly, imaging-based grading of fibrosis has been included in clinical guidelines and recommendations [7, 8, 9, 10]. Finally, imaging is an important part of evaluating patients for liver transplantation, representing the only curative treatment option for children and adolescents with end-stage liver disease.

## Detection of cirrhosis

Due to its widespread availability and noninvasiveness, abdominal ultrasound (US) is the primary imaging tool in children to diagnose diffuse liver disease. The US examination is often demanded at a very basic level during the clarification process with the purpose of screening patients for possible abnormalities. Therefore, familiarity with the common US signs of chronic liver damage is important.

To maximize the diagnostic capacity, all available US techniques and the full spectrum of probes should be applied. Today, most US systems are equipped with lower-resolution convex probes, high-resolution linear probes, various vascular imaging techniques (i.e. color Doppler) and elastography, allowing a multiparametric approach [11]. Some manufacturers also suggest evaluating steatosis with an attenuation imaging technique. The US examination should cover the entire abdomen and follow a systematic approach with a detailed assessment of the liver (including intra- and extrahepatic bile ducts, gallbladder), pancreas, spleen (with length measurement), vessels and bowel and mesentery [12]. Children with cirrhosis are at risk of developing extrahepatic manifestations of portal hypertension (including splenomegaly, ascites, bowel wall thickening, varices and portosystemic shunts).

Magnetic resonance imaging (MRI) and computed tomography (CT) are considered second-line modalities in children, which are reserved to answer specific questions during work-up. Younger children’s difficulty with cooperating, their inability to lay still for long periods, their inability to breath-hold and their overall smaller body size, with delicate anatomical structures, make cross-sectional imaging with MRI and CT generally more challenging and as such, they require tailored protocols [13]. Moreover, children younger than 6 years old and children with disabilities often require sedation to limit movement.

## Morphological changes

Morphological alterations affect hepatic size, shape and texture. These changes can be detected with all available cross-sectional imaging modalities including US, MRI and CT and the general features of liver cirrhosis are very similar in children and adults [2].

With US, assessment of parenchymal integrity is based on the grey-scale B-mode technique. Hepatic vasculature is differentiated anatomically from the biliary system by using color Doppler, among other vascular imaging techniques. As a rule, convex transducers are used first as they give a good overview and have sufficient penetration to cover the entire organ. High-resolution linear US probes (typically up to 15 MHz) should be used in addition, as they offer better spatial resolution. Their application is especially beneficial in neonates with small anatomical structures (common bile duct 1–2 mm) [12]. For this age group, the differentiation between obstructive and nonobstructive neonatal cholestasis, especially between biliary atresia and neonatal hepatitis, mainly relies on US [14]. The triangular cord sign, an abnormal gallbladder, micro- or macrocysts at the porta hepatis and polysplenia are specific findings associated with biliary atresia [15]. However, a normal US examination does not exclude biliary atresia.

**Fig. 1:**
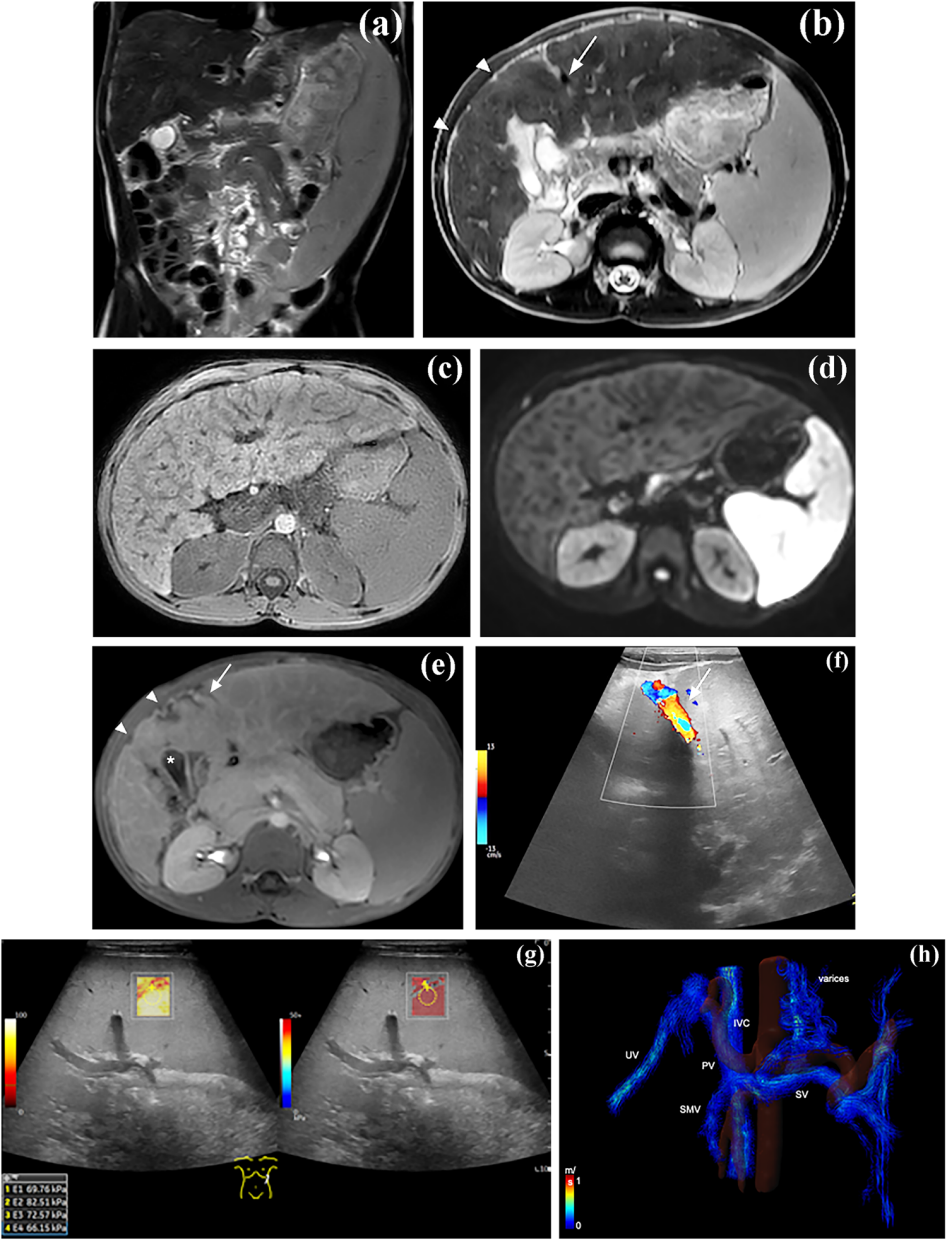
A 9-year-old girl with nephronophthisis (NPHP3 gene mutation) and congenital hepatic fibrosis. Massive splenomegaly can be noted on the coronal T2-TSE (**a**). The liver has diffuse textural changes depicted in all standard axial sequences (**b**, T2-W; **c**, T1-W; **d**, diffusion weighted imaging, b800; **e**, contrast-enhanced T1-W). Hypertrophy of segment 2/3, hypotrophy of the right lobe, enlargement of the pericholecystic space (*asterisk*) and capsular retraction (*arrowheads*) can be seen. Recanalization of the umbilical vein is noted on standard magnetic resonance imaging (MRI) sequences (*arrow*), but more clearly depicted on transverse color Doppler US (**f**). On US elastography (**g**, linear probe in longitudinal plane, confidence map on the right, elastogram on the left) spleen stiffness was substantially increased (median 2-D shear wave elastography values, 70 kPa) suggestive of congestion by portal hypertension. Grade 3 esophageal varices were noted on endoscopy (not shown). Four-dimensional flow MRI has been used to fully visualize the portosystemic communications (**h**, coronal reconstruction; *ivc* inferior vena cava, *pv* portal vein, *smv* superior mesenteric vein, *sv* splenic vein, *uv* umbilical vein)

**Fig. 2:**
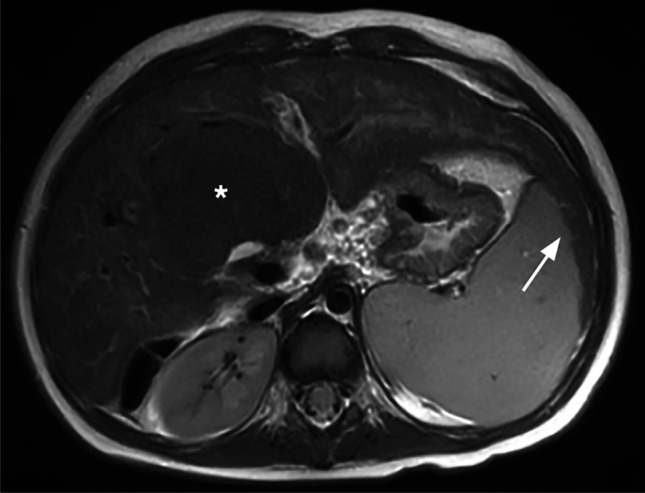
A 2-year-old girl with biliary atresia after a Kasai operation. On axial T2-W magnetic resonance imaging, segmental hypertrophy of liver segment 1 with the formation of a regenerative nodule (*asterisk*), as well as enlargement of liver segment 2/3 with kissing sign of liver and spleen (*arrow*), can be noted

The extent of the common morphological changes in liver cirrhosis depends on the stage and type of the disease. In early, compensated cirrhosis, many patients show hepatomegaly [16, 17]. Additional early signs include widening of the porta hepatis, enlargement of the interlobar fissures and expansion of the pericholecystic space (Fig. 1) [18]. Later in the course, more extensive fibrosis and overall volume loss may be noted [16]. Typical is a side-to-side finding of lobar hypotrophy (e.g., of the right liver lobe plus the medial part of segment 4) and lobar hypertrophy of the left lateral lobe (segment 2 and 3), extending the liver toward the left. With left lateral lobe hypertrophy a “kissing” phenomenon with the spleen is described (Fig. 2) [19].

**Fig. 3:**
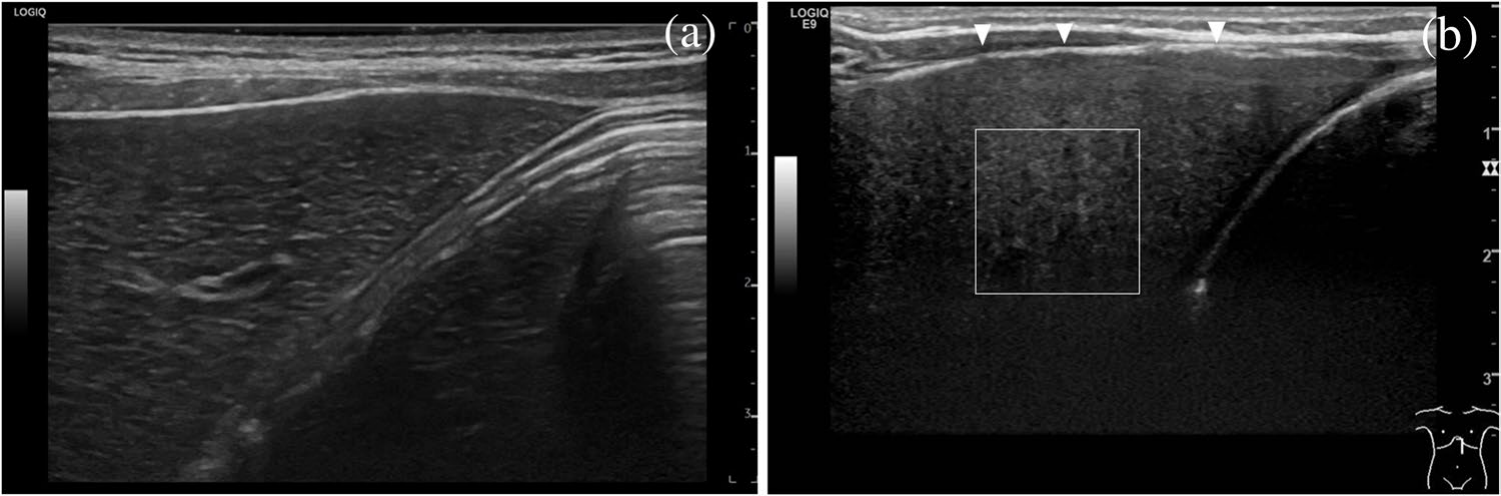
Longitudinal left subcostal ultrasound for the evaluation of liver texture using a linear probe. Regular liver texture in a 5-year-old healthy boy shows a homogenous distribution with relatively low differences in echogenicity between neighboring pixels (**a**). The liver contour is smooth. By comparison, the irregular appearance of the liver texture is shown in a 3-month old boy with liver cirrhosis due to biliary atresia (**b**). Note the wider distribution of grey-scale values and the increasing beam attenuation with image depth. The liver contour is nodular (*arrowheads*)

The left lobe of the liver at the left subcostal border is a good position to look for abnormalities in shape and contour. A healthy liver is characterized by a smooth, even surface and a pointed lower edge (Fig. 3). With progressive hepatic remodeling, loss of shape and nodular rounding of the liver border occurs. Confluent fibrosis is accompanied by capsular retraction [20, 21, 22]. Macronodular changes affecting the liver contour are well depictable, whereas lower degree micronodular changes may be missed. High-resolution linear US transducers should be used for additional evaluation of the liver surface. Identifying fine nodular irregularities at the superficial boundary of the left lobe is a specific finding in cirrhosis that can be used to rule-in cirrhosis in doubtful cases and has prognostic implications in adults [23, 24].

**Fig. 4:**
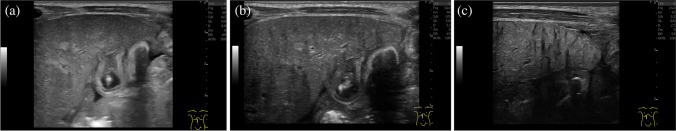
Longitudinal left subcostal ultrasound of the left liver lobe in a 10-week-old girl with biliary atresia, who developed progressive fibrosis despite a Kasai procedure. The appearance of textural changes of the left liver lobe depends on the choice of linear transducer and the selected frequencies. Textural changes are more distinct with lower frequency transducers (**a**, 9 Mhz; **b**, 14 Mhz). Fibrotic bands are visible on the highest-resolution images (**c**, 24 Mhz), but beam attenuation notably decreases penetration and visualization of deeper structures

Conventional US also notes texture, which is a qualitative description of the liver parenchyma. Whereas a normal hepatic texture has a uniform and homogeneous appearance on US, a liver altered by fibrosis shows increased echogenicity with uneven distribution between neighboring pixels as well as higher beam attenuation. Textural changes may appear as a general coarseness or roughness (Fig. 3). The depiction of textural changes depends on the equipment and is facilitated by using higher resolution transducers which are helpful for additional evaluation (Fig. 4).

Overall, one must be cautious to diagnose or exclude cirrhosis based on morphological signs only. For instance, textural changes are not fully specific and steatosis can mimic the appearance of fibrosis, showing a fatty-fibrotic pattern [25, 26]. Also, the sensitivity of conventional US to pick up cirrhosis or high-grade fibrosis by screening for morphological abnormalities is relatively low, as shown in a meta-analysis [27].

## Refined tissue characterization

### Automatic texture analyses

Researchers have attempted to overcome user dependency on descriptive visual grading by developing automatic methods of categorization and quantification of textural abnormalities using computer-aided analysis and neuronal networks. Distribution of grey-scale values and differences in US echogenicity within regions of interest have been analyzed by different mathematical models and correlated with the histological fibrosis stage [28, 29, 30]. Automated texture analysis has also been performed on standard MRI sequences (including diffusion-weighted imaging [DWI], susceptibility-weighted imaging, T2-W and contrast-enhanced T1-W imaging) [31, 32, 33] and for CT [34]. Automated scoring of surface nodularity on CT was able to detect patients with cirrhosis and was a predictor for decompensation and death [24]. However, for most pediatric radiologists, these techniques have limited accessibility in daily practice.

### Diffusion-weighted imaging and mapping techniques

Diffusion-weighted imaging can depict areas of focal and diffuse fibrosis and has been shown to be very sensitive for the detection of focal liver lesions [35]. Therefore, DWI is included in standard protocols for liver imaging (Fig. 1). Quantification of diffusivity can be performed by measuring the apparent diffusion coefficient (ADC). ADC values are lower in patients with advanced fibrosis due to restricted diffusion of water molecules [36] and studies have been published using ADC values to grade fibrosis [37]. However, ADC values depend on scanner-specific presets (with problems of standardization) and are influenced by several confounders (including steatosis and iron deposition) making them an unreliable tool for routine clinical grading of liver fibrosis in children. A recent study in adults introduced a method to correct ADC values by additionally measuring the fat fraction and demonstrated improved detection of significant fibrosis when coexisting with steatosis [38]. T1, T2 and T1rho mapping have also been used in research to characterize tissue changes in cirrhosis. Relaxation times increase with fibrosis and edema [39]. However, further pediatric studies are needed to standardize setups, to define normal values and to evaluate performance in specific disease entities.

### Tissue composition (fat fraction and iron load)

Quantitative methods to analyze tissue composition are used to measure liver fat content and iron deposition in children. Steatosis often coexists with fibrosis and occurs in connection with viral infections, metabolic disorders (e.g., Wilson disease) and in NAFLD [40]. NALFD has been described in up to 8% of the pediatric population and can result in fibrosis or even cirrhosis in late childhood [41]. Chemical shift-encoded MRI is the most accurate method to measure fat content [42]. A fat fraction map of the liver can be generated within a single breath-hold. For children and patients who are incapable of breath-holding, free-breathing methods have been published [43, 44]. Steatosis can also be visually graded on conventional B-mode US (grades 1–3, mild-moderate), but the overall performance (especially for low-grade steatosis) has been reported to be relatively low [45]. Promising new applications are quantitative US methods, which different vendors have included on their US platforms and which can determine the amount of liver fat at the bedside. However, the variety of US techniques complicates comparability. The best applications and their performance in children of different age groups and with different disease entities are yet to be defined [45].

Severe liver damage in a newborn accompanied by extrahepatic siderosis is a sign of neonatal hemochromatosis and mainly attributable to gestational alloimmune liver disease (GALD). In GALD, maternal antibodies are directed against a protein specifically expressed on fetal hepatocytes causing hepatocyte loss, panlobular fibrosis and formation of regenerative nodules [46, 47]. The pattern of extrahepatic iron deposition in organs outside the reticular endothelial system (e.g., pancreas, salivary glands, thyroid) is similar to hereditary hemochromatosis. MRI can quantitatively or semiquantitatively measure extrahepatic siderosis and has a role in the diagnostic work-up of neonates with suspected GALD before treatment with exchange transfusion and intravenous immunoglobulin is initiated [48]. Buccal biopsy or liver biopsy can be risky due to coagulopathy and potential bleeding. The finding of hepatic iron overload is relatively nonspecific. It is a feature of hemochromatosis but can be frequently encountered in various forms of advanced liver disease, including chronic hepatitis C virus infection and NAFLD [49].

## Hemodynamics and portosystemic communications

Progressive hepatic remodeling during liver cirrhosis affects hepatic perfusion and the specific evaluation of hepatic hemodynamics is part of the diagnostic work-up. Color Doppler is the primary US-based imaging technique used to depict intra- and extrahepatic vessels, in which spectral waveform analysis is applied to assess flow velocity and direction. US often better provides dynamic information on the small hepatic vasculature than MRI or CT, especially in younger children [50].

Portal venous flow directed from the splenic and mesenteric axis into the liver is a low-pressure, low-gradient system that is very sensitive to parenchymal changes resulting in increased organ resistance. Children with significant liver fibrosis typically show a subsequent reduction of total liver flow [51]. The portal flow velocity is reduced compared to non-cirrhotic patients and can be reversed in advanced stages [52]. Portal vein hypoplasia (≤ 4 mm) is regularly observed in children with biliary atresia-associated cirrhosis, many of them needing portal reconstruction in the form of liver transplantation [53]. Liver cirrhosis is also a risk factor for portal vein thrombosis.

Reduced portal venous inflow is physiologically compensated for by an increase in arterial inflow to sustain organ perfusion (hepatic buffer response). It is a typical finding in young children with biliary atresia-associated progressive liver fibrosis, displaying high peak systolic velocities, an increased resistive index (RI > 0.9) and hypertrophy of the hepatic artery [14, 51]. As a result of reduced elasticity in fibrosis, pressure changes of the right atrium are less well transmitted into the liver veins. Liver fibrosis is associated with a loss of phasic modulation of the hepatic venous outflow. Instead of a triphasic flow pattern, patients with liver cirrhosis may often display a biphasic flow with low modulation or flat monophasic outflow [54]. Hepatic outflow modulation can be measured by calculating a hepatic waveform index. Biphasic and low modulation flow can also be a normal finding. Altogether, assessment of hepatic inflow and outflow by US has not been shown to be a sensitive predictor of the degree of liver fibrosis and therefore should not be used for staging [52, 55].

### Portosystemic communications

**Fig. 5:**
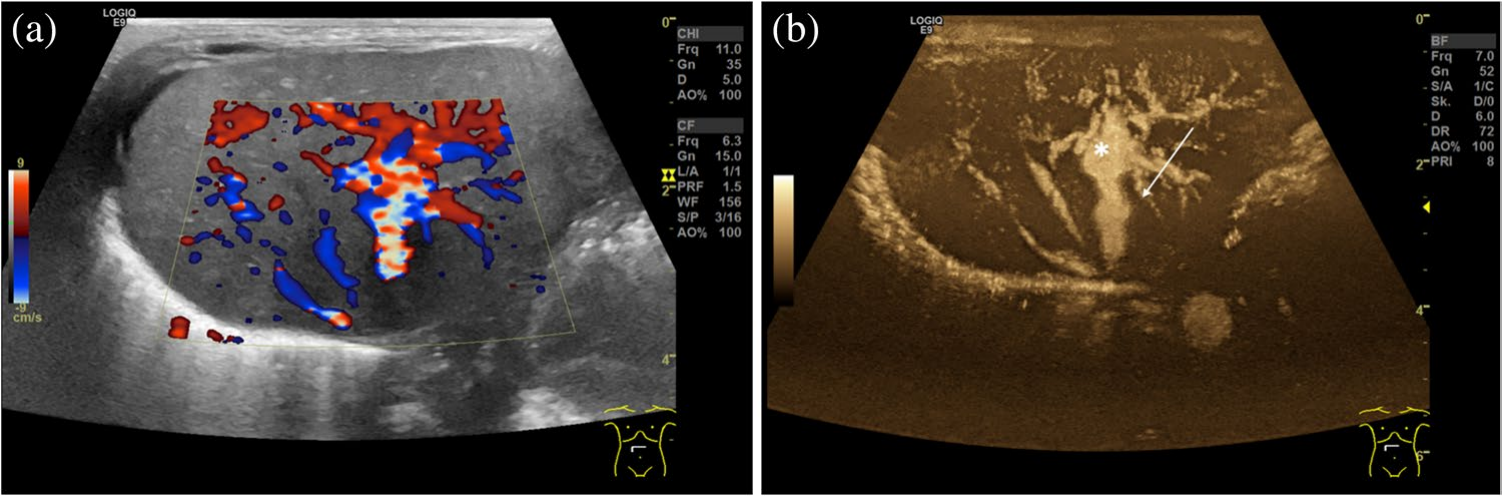
Ultrasound in a 10-day-old boy with atypical Shwachman-Diamond-like syndrome complicated by liver cirrhosis. Corresponding axial images at the level of the liver veins in color Doppler (**a**) and b-flow (**b**) show a large, persistent ductus venosus (*arrow*) functioning as an intrahepatic portosystemic shunt. Umbilical recess (*asterisk*)

Portosystemic shunting frequently occurs in patients with progressive liver fibrosis and is a reaction to the increased intrahepatic pressure gradient. Blood from the splenic and mesenteric circulation bypasses the liver by intrahepatic or extrahepatic portosystemic communications [19]. A re-opened umbilical vein draining blood from the umbilical recess to the systemic circulation can be frequently encountered in children of all ages and is sensitively detected by US as there is no superimposition of gas. The umbilical vein runs superficially within the falciform ligament and can be identified as a tubular structure showing a monophasic hepatofugal flow (Fig. 1). A persisting ductus venosus is typically seen in neonatal hemochromatosis and other forms of diffuse neonatal liver disease [56]. The ductus venosus is part of the fetal circulation, carrying blood from the umbilical vein via the portal recess to the venous confluence and normally closes shortly after birth (< 1 month in premature babies and < 2 weeks at term) [57]. The ductus venosus can be identified by color Doppler US (showing a high velocity hepatofugal flow pattern), as well as with other cross-sectional imaging modalities (Fig. 5). Extrahepatic portosystemic shunts may be difficult to depict with US due to their complexity and superimposition of bowel gas. Thickness of the lesser omentum and, more specifically, the left gastric vein (with size and direction of flow) should be assessed with a convex transducer, while perigastric and perisplenic varices can be depicted with a high-frequency probe. For full delineation of abdominal vessels and identification of atypical communications at intra- or extrahepatic sites, contrast-enhanced CT or MRI with multiplanar reconstructions are often superior [2, 58]. Four-dimensional flow MRI (4-D flow MRI) can be used for functional analyses without the use of contrast. Four-dimensional flow MRI is a relatively new 3-D, time-resolved phase contrast imaging technique that enables visualization and quantification of blood flow in the abdominal vasculature during a single examination [59] (Fig. 1). The technique has been tested in a feasibility study of adults with cirrhosis to stratify their risk for bleeding from esophageal varices [60]. Although in one study, direct identification of esophageal varices succeeded only in a few patients, a disproportionate portal venous flow (portal venous flow < sum of the flow in superior mesenteric vein and splenic vein) showed good performance in the prediction of esophageal varices at high risk of bleeding (sensitivity 100%, specificity 94%). Another study found a postprandial increase in flow rate in the azygos vein, which is known to be associated with esophageal varices [61, 62].

## Liver stiffness

Noninvasive stiffness imaging techniques have been developed that can depict and quantify tissue elasticity in children. Ultrasound-based methods comprise vibration-controlled transient elastography (VCTE) and acoustic radiation force impulse (ARFI)-based techniques with point shear wave elastography (pSWE) and 2-D shear wave elastography (2-D SWE). VCTE is available on a stand-alone, non-imaging-based system using a mechanical push to induce a shear wave [63]. ARFI techniques have been implemented by most vendors directly on their US platforms with the advantage that liver stiffness can be measured as part of a routine clinical US examination.

Magnetic resonance elastography (MRE) uses an active-passive mechanical driver system placed on the right upper abdomen to generate shear waves in the liver at a defined frequency [64]. Shear wave propagation can be imaged by MRI using a modified phase-contrast pulse sequence that is converted to an elastogram [65]. In contrast to the US methods, cross-sectional MR elastograms can cover the entire liver, which is advantageous the liver is not homogeneously affected by fibrosis, as it reduces sampling error [66]. MRE is less widely available than US elastography, to perform MRE, extra equipment is necessary and children younger than 6 years may require sedation [64].

Assessment of liver stiffness in younger children and infants is often complicated by low cooperation and movement. Therefore, it is important to generate a child-friendly atmosphere, e.g., to examine the child on a parent’s arm [67]. However, deep inspiration and liver displacement during free respiration can cause artifacts that cannot be easily controlled by the breath-hold maneuver as recommended for adults [68]. For practical reasons, studies have adopted a free-breathing approach for noncooperative pediatric patients [69]. To obtain meaningful results, it is recommended that a qualitative map is used to select the most accurate samples and to discard elastograms showing artifacts due to gross movement. Higher-frequency linear probes can produce more reliable results in infants and young children, but the values tend to be higher compared to lower frequency convex transducers, as the shear wave speed depends on the ARFI frequency [70].

### Normal values

All US-based methods and MRE are principally applicable for use in children including infants. Reported mean values in healthy children have been published for the different US-based methods and for the platforms of different vendors (ranging between 3.4 and 6.58 kPa) [71, 72, 73, 74, 75, 76]. Overall, normal values vary slightly when equipment from different vendors, different US techniques or different probes are applied [77, 78]. In clinical practice, this factor seems to be especially relevant for individual follow-up of patients, which should be performed with a similar setup as the baseline examination to reduce spurious variability in results. A slight age dependency of liver elasticity has been shown using VCTE (3.4 kPa in ages 1–5 years to 4.1 kPa in ages 12–18 years) [79] and 2-D SWE (4.9 kPA in ages 3–6 years and 5.2 kPa in ages 12–18 years [80]. A study including 128 healthy children showed that height was the only significant predictor of shear wave speed (positively correlated) [74]. The reported values in kPa for MRE are about three times smaller than those reported for US techniques based on Young’s modulus [9]. In one study, mean stiffness in 81 healthy children ages 8 to 17 years was 2.45 ± 0.35 kPa (95th percentile 3.19 kPa), showing higher values than in adults (2.10 ± 0.23 kPa) [81]. In another study with similar age range in 71 children, the mean liver stiffness was 2.1 kPa [81, 82].

### Staging of liver fibrosis

Ultrasound elastography as well as MRE have been applied to stage liver fibrosis. A meta-analysis looking at the performance of VCTE in 723 children in 11 studies showed 95% sensitivity and 90% specificity to detect significant fibrosis (≥ F2 METAVIR, cutoff value 10.6 kPa) [83]. Another meta-analysis summarized results in 550 children using ARFI for staging significant fibrosis and found a sensitivity of 81% and specificity of 91% (2-D-SWE, median cutoff value 9.4 kPa). Two-dimensional SWE performed significantly better than pSWE [38]. A comparable performance has been reported for MRE, differentiating between early and late-stage hepatic fibrosis. One study found 88% sensitivity and 85% specificity for the detection of significant fibrosis in children (cutoff value 2.7 kPA) [84]. A meta-analysis summarizing MRE in 697 adults found an overall sensitivity of 88% for detecting fibrosis grade ≥ F2, which was independent of the underlying etiology [85].

Elastography has also been assessed as a diagnostic tool for specific disorders. In neonatal cholestasis, US elastography can assist in differentiating biliary atresia from other cholestasis disorders not requiring early surgery. A VCTE study evaluating 48 neonates with cholestasis showed higher values for biliary atresia than for infants of the same age with other cholestasis syndromes (80% sensitivity and 97% specificity, cutoff value > 7.7 kPa) [86]. Similar studies applied ARFI-based methods (pSWE and 2-D SWE) and confirmed that higher stiffness values are in support of biliary atresia and may facilitate earlier diagnosis in unclear cases [87, 88].

However, it is important to acknowledge that elevated liver stiffness is not an exclusive marker of fibrosis but can be caused by other factors such as hepatic congestion, inflammation, cellular infiltration or deposition of metabolites [9]. In many clinical circumstances, increased liver stiffness may be a summation of multiple factors. For instance, young children after stage 3 completion of Fontan surgery for single ventricle repair showed an immediate increase in liver stiffness values that was attributable to liver congestion due to increased central venous pressures [89]. As chronic elevation of central venous pressure can induce fibrosis in the long term, older patients after Fontan surgery exhibit higher stiffness values that may be related to fibrosis and congestion. A follow-up study showed relatively high median SWE values in Fontan patients compared with controls (15.6 vs. 5.5 kPa). Numbers in Fontan patients were substantially increased when significant fibrosis was present (19.8 vs. 13.4 kPa) [9, 90]. The link between liver congestion and increased liver stiffness has also been demonstrated in Budd-Chiari syndrome and liver vein stenosis after liver transplantation [91, 92].

## Assessment of disease evolution and complications during follow-up

Medical imaging is regularly applied to monitor children with chronic liver disease. The choice of modality and imaging frequency depends on various factors and is part of clinical decision-making. Important determinants are the underlying disease-associated risk profile, the interval before introduction of treatment and the presence of liver-related compensation with signs of decompensation (ascites, significant jaundice, hepatic encephalopathy, variceal bleeding, portal vein thrombosis and hepatocellular carcinoma [HCC]). Children with cirrhosis and lower risk profile and stable clinical performance are seen every 6–12 months, in case of concern, intervals will be shortened.

US is the main modality for chronic liver disease follow-up in children as it can be repeated frequently without harm and changes can be identified by comparing to baseline examinations. To obtain meaningful results, it is necessary to apply strict standardization methods. For morphological and functional assessment, a set of defined image planes through the liver is documented, representing key structures including the left lobe at the left subcostal border, gallbladder and (patent) portal vein. Examination also includes color Doppler measurements of hepatic vessels, measurement of organ sizes including spleen and elastography.

**Fig. 6:**
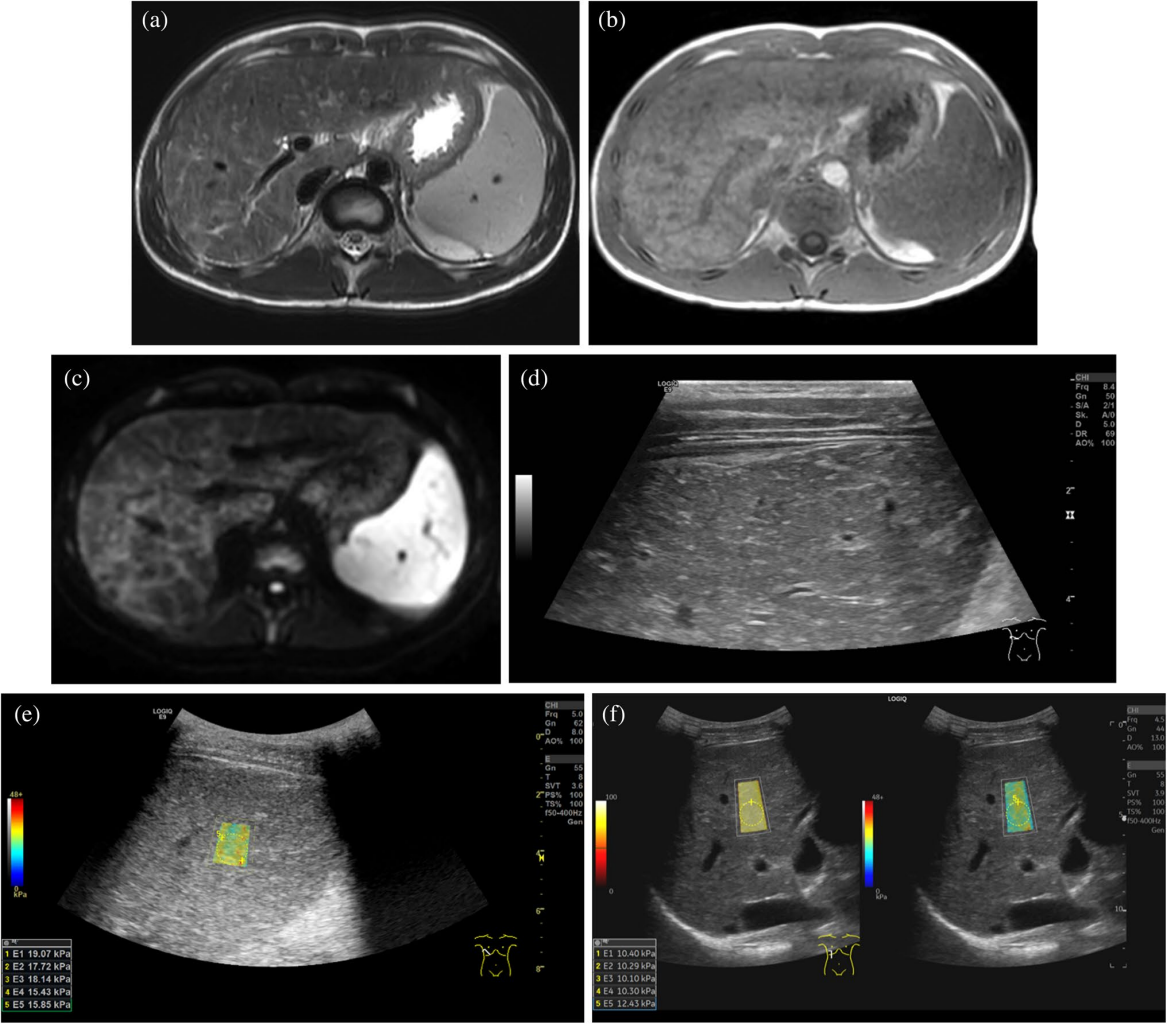
A 13-year-old boy with autoimmune hepatitis and liver cirrhosis. Textural changes can be noted on axial magnetic resonance images of the liver on T2 turbo spin echo (**a**), T1 mDixon (**b**) and the diffusion-weighted b800 image (**c**). Areas of diffusion restriction in the right lobe represent fibrosis. Textural changes can also be noted on gray-scale right intercostal ultrasound (**d**). Right intercostal US with a convex probe, 2-D shear wave elastography (2-D SWE) at the time of diagnosis (**e**) and 12 months after start of immunosuppressant treatment under complete biochemical remission (**f**, confidence map, left and elastogram, right). The reduction in liver stiffness (delta of median 2-D SWE values, -6.5 kPa) is explained by elimination of the inflammatory component. The persisting elevation of liver stiffness at 12 months indicates a remaining fibrotic component

Quantitative longitudinal measurement of liver stiffness is used to control disease activity in a variety of disorders. The change in liver stiffness over time (delta) has been suggested to direct management of patients with Fontan circulation [93, 94]. Liver stiffness has been established as a quantitative, noninvasive method to monitor disease progression in patients with autoimmune disorders and especially autoimmune hepatitis [95]. In autoimmune hepatitis, by the time a liver biopsy is performed, it shows autoimmune cellular infiltrates and in many cases progressive fibrotic change. Within 6 months after introducing an effective immunosuppressant treatment, the reduction in stiffness values is explained by the elimination of inflammatory change (Fig. 6). With permanent biochemical remission, a further reduction in liver stiffness has been linked to a regression of fibrosis, which was verified by follow-up biopsies in adults. Hartl et al. [95] have recommended yearly US examinations to detect patients who have mild residual disease activity despite full biochemical remission and who may potentially have an adverse long-time outcome [96].

### Portal hypertension

The evolution from compensated liver cirrhosis to a decompensated state is linked to the development of portal hypertension. Architectural distortion of the liver results in increased resistance to blood flow through the hepatic sinusoids. Clinically significant portal hypertension (CSPH) is defined as an increase in the portal venous pressure gradient (HVPG) above 10 mmHg and has been linked to an increased risk for the development of esophageal varices, decompensation and hepatocellular carcinoma in adults [97]. As HVPG measurement is invasive, it is rarely performed in children. Yet, conventional imaging signs that may be present in portal hypertension, such as splenomegaly, portosystemic collaterals or ascites, are not closely linked to the CSPH and esophageal varices.

In adults, measurement of liver and spleen stiffness have been included in an algorithm to stratify the risk of CSPH and consequently variceal bleeding. Patients with a liver stiffness < 20 kPa on VCTE and a platelet count > 150,000 are at low risk for variceal bleeding (Baveno VI criteria) [98]. In case of thrombocytopenia or increased liver stiffness, patients with spleen stiffness > 46 kPa should undergo esophagogastroduodenoscopy. Spleen stiffness may be a better indicator of portal hypertension than liver stiffness (Fig. 1). A pediatric study of 34 children with biliary atresia after the Kasai procedure found spleen stiffness the best predictor for esophageal varices (2-D SWE values > 4.12 m/s, sensitivity 92.9%, specificity 90%) [99]. Another pediatric study using VCTE in chronic diffuse liver disease (*n* = 52) and portal vein thrombosis (*n* = 15) demonstrated that spleen stiffness was superior to liver stiffness for predicting high-risk gastroesophageal varices (sensitivity 77% vs. 67%, specificity both 87%) [100]. Further studies are needed in children to clarify the diagnostic capacity of liver and spleen stiffness measurements for noninvasive detection of portal hypertension.

### Hepatocellular carcinoma

**Fig. 7:**
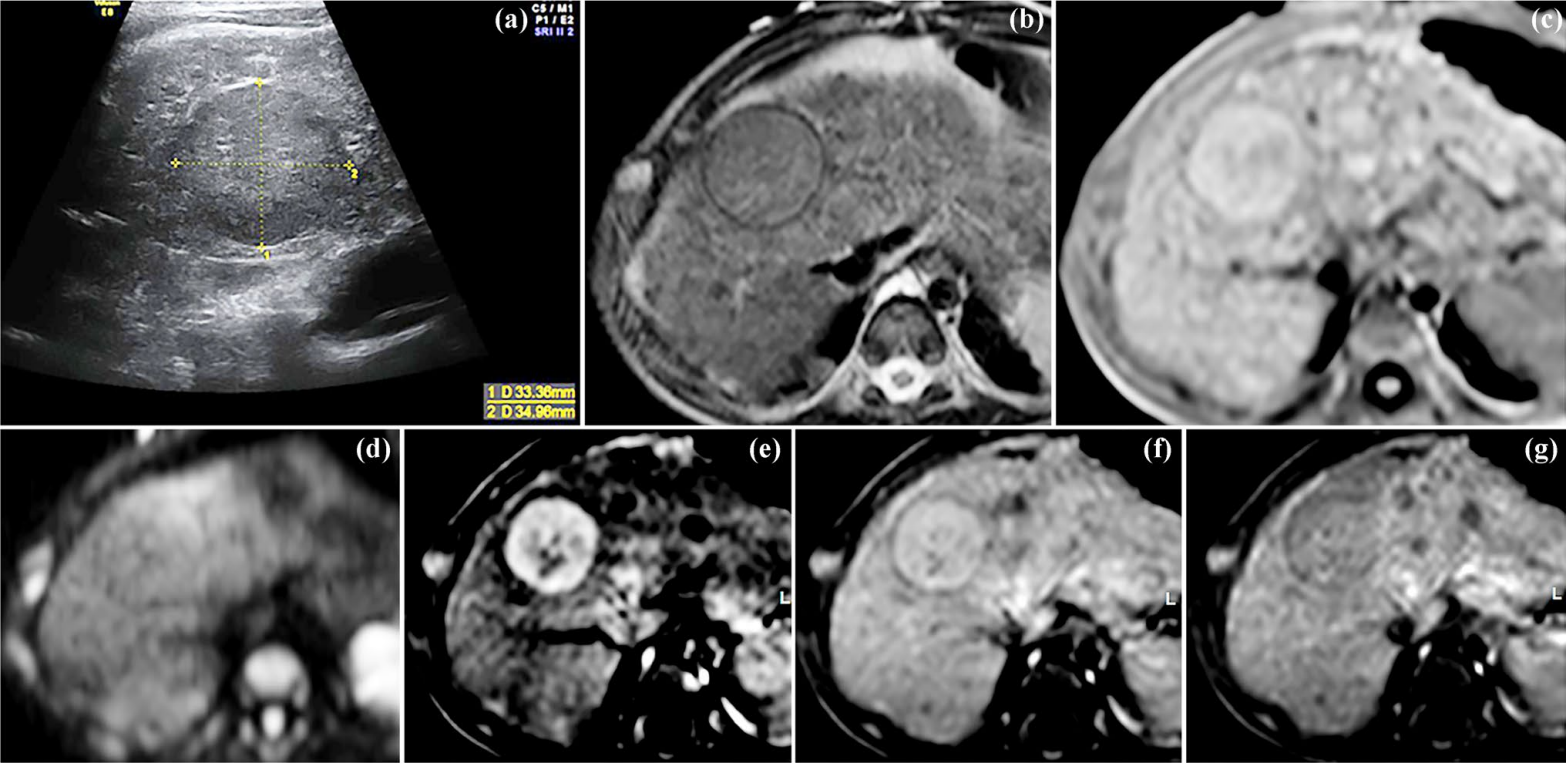
A 2-year-old boy with hereditary tyrosinemia type 1 and hepatocellular carcinoma. Right intercostal ultrasound (**a**) shows an echogenic lesion within an irregular hepatic texture. The lesion is isointense on axial T2 turbo spin echo (**b**), hyperintense on axial T1 (**c**) and only diffusely demarcated on axial diffusion-weighted imaging (b800 image, **d**). After contrast administration, the lesion shows strong enhancement on the axial T1 in the arterial (**e**) and portal-venous phase (**f**) and slight washout in the parenchymal phase (**g**)

Development of nodular lesions is a key feature of cirrhosis. These lesions can be classified as regenerative nodules, dysplastic nodules and HCC (Figs. 2 and 7) [19]. HCC is, after hepatoblastoma, the second-most-frequent primary malignant liver tumor in children. In about 25–40% of children, HCC is linked to progression of an underlying chronic liver disorder (cholestatic, metabolic and viral diseases as well as cardiac cirrhosis) [1]. For instance, children with biliary atresia treated successfully with the Kasai operation often develop cirrhosis during follow-up and HCC as well as cholangiocarcinoma have been sporadically detected with increasing age [101, 102]. Other inherited cholestatic disorders associated with HCC in childhood are Alagille syndrome, progressive familial intrahepatic cholestasis (PFIC type 2 and bile salt pump deficiency; HCC at age 2 years is seen in 5–10% with PFIC type 2) [103]. Children with hereditary tyrosinemia type 1 (fumarylacetoacetase deficiency), if untreated from the first weeks of life, are at very high risk of developing HCC in early childhood [104] (Fig. 7). Glycogen storage disease type 1 (glucose-6-phosphatase deficiency) increases the risk of adenomas with malignant transformation after puberty [105].

Depending on the underlying disorder, abdominal US every 6 to 12 months together with measurement of alpha-fetoprotein (AFP) has been suggested for children at risk [106, 107]. In experienced hands, US has been shown to have relatively high sensitivity for detecting HCC in patients with cirrhosis. However, HCC lesions can be hypo- or hyperechogenic compared to the surrounding liver and reliable differentiation between regenerative nodules, dysplastic nodules and HCC is not possible with conventional US [108]. Increasing AFP levels suggest HCC. However, normal or unchanged AFP values in the absence of a new liver lesion on US do not exclude HCC [109, 110].

Imaging should follow the consensus imaging guidelines for children with hepatic neoplasms that have been published by the pediatric liver reporting and data system (LI-RADS) working group [111, 112]. Abdominal US is considered the primary imaging modality and is used for screening and follow-up of liver lesions. If a focal lesion is detected, contrast-enhanced US (CEUS) is suggested to further assess the lesion’s potential for malignancy. MRI should be performed if more than two lesions are discovered or the chance for malignancy is high on initial US. As a specific hepatocyte phase can identify additional lesions and contribute to improved risk stratification [113, 114], the consensus guideline strongly recommends hepatobiliary contrast agents (HBA: gadoxetate disodium, GdEOB-DTPA; Eovist/Primovist, Bayer, Germany; gadopentetate dimeglumine, Gd-BOPTA; MultiHance, Bracco, Italy) for initial clarification and during follow-up at every time point [111]. It is important to note that HBA in children may be used off-label as these contrast agents have not been officially approved in several countries.

Due to the increased risk of ionizing radiation, CT is mainly advocated for extended staging to exclude lung metastasis or when MRI is not possible. CT is faster than MRI, has excellent spatial resolution and has advantages in depicting vascular anatomy before surgery and before liver transplantation [115]. If surgical resection of HCC is not possible, liver transplantation has been shown to be a curative option, with a 5-year disease-free survival of 84.5% [116].
